# Supplementary material for: Integrated analysis of the aqueous humor microbiome and lens capsule transcriptome in high myopia cataract: a pilot study
Source: Front Med (Lausanne). 2026 Jun 16;13:1845205. doi: 10.3389/fmed.2026.1845205 (PMC13314463; doi:10.3389/fmed.2026.1845205)
Supplement: Supplementary file 3 [file Data_Sheet_1.zip › 3.Beta_Diversity/UPGMA/C372089/binary_jaccard_circular_Tree.pdf]

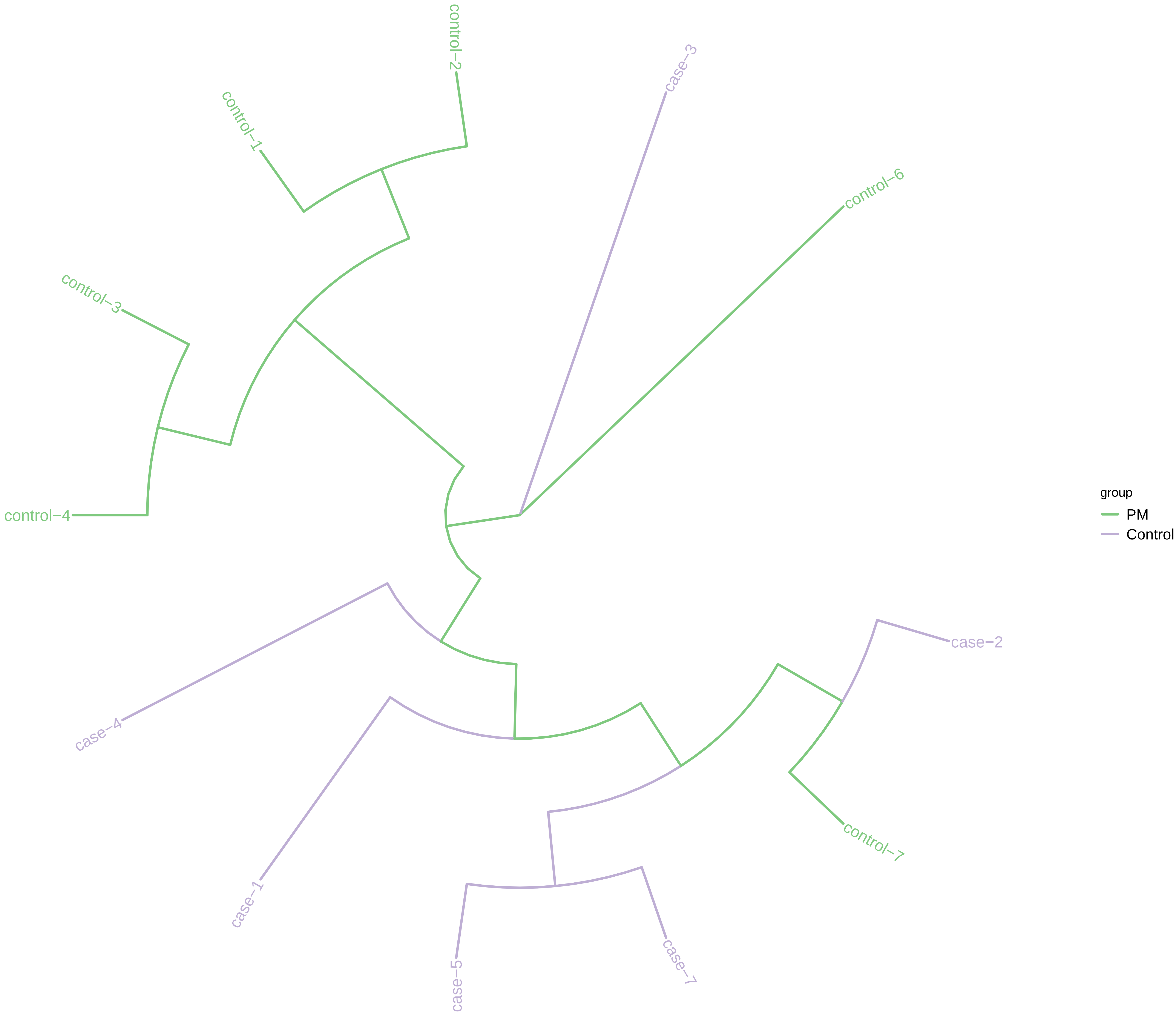
control−2
case−3
control−1
control−6
control−3
control−4
case−2
case−4
control−7
case−1
case−5
case−7
group
PM
Control
